# Quality of Life Is Related to Fecal Calprotectin Concentrations in Colonic Crohn Disease and Ulcerative Colitis, but not in Ileal Crohn Disease: Erratum

**DOI:** 10.1097/01.md.0000489591.67334.e9

**Published:** 2016-07-18

In the article “Quality of Life Is Related to Fecal Calprotectin Concentrations in Colonic Crohn Disease and Ulcerative Colitis, but not in Ileal Crohn Disease”,^1^ which appeared in Volume 95, Issue 16 of *Medicine*, Dr. Geiss's name was incorrectly spelled as Geib. The article has since been corrected online.

## References

[1] Gauss A Geib T Hinz U Quality of life is related to fecal calprotectin concentrations in colonic Crohn disease and ulcerative colitis, but not in ileal Crohn disease. *Medicine.* 2016 95:e3477. 27100452 10.1097/MD.0000000000003477 PMC4845856

